# Effects of punctate skin grafting combined with or without irrigation on skin graft survival, redness and swelling score and pain in treatment of large-area residual burn wounds

**DOI:** 10.12669/pjms.38.7.5522

**Published:** 2022

**Authors:** Lei Wan, Jin Zhou, Linjie Li

**Affiliations:** 1 Lei Wan, Department of Critical Care Medicine, Huangshi Central Hospital, Affiliated Hospital of Hubei Polytechnic University, Edong Healthcare Group, Huangshi 435000, Hubei, P.R. China; 2 Jin Zhou, The Central Hospital of Xiaogan Emergency Surgery, Xiaogan 432100, Hubei, P.R. China; 3 Linjie Li, Department of Dermatology, Traditional Chinese Medicine Hospital, Edong Medical Group, Huangshi 435000, Hubei, P.R. China

**Keywords:** Punctate skin grafting, Irrigation, Large-area residual burn wound, Skin graft survival, redness and swelling score, Pain score

## Abstract

**Objective:**

To investigate the effects of punctate skin grafting combined with or without irrigation on skin graft survival, redness and swelling score and pain in the treatment of large-area residual burn wounds.

**Methods:**

A prospective study was conducted on 102 patients with large-area burns treated in Huangshi Central Hospital from May 2017 to May 2020. According to different treatment methods, they were divided into combination group (treated with punctate skin grafting combined with irrigation) and grafting group (mainly treated with punctate skin grafting). The positive rate of bacterial culture of wound secretions, pain score and redness and swelling score were analyzed and compared between the two groups. Additionally, linear logistic analysis of the influencing factors was carried out.

**Results:**

The positive rate of bacterial culture of wound secretions decreased to 29.4% in the combination group and 49.0% in the grafting group (p< 0.05). The skin graft survival rate of the combination group was (82.17 ± 7.44) %, which was higher than that of the grafting group (61.53 ± 7.46) %, the wound healing time was (14.56 ± 3.51) d, which was shorter than that of the grafting group (21.36 ± 4.69) d, and the pain score (3.26 ± 0.36) and redness and swelling score (1.16 ± 0.68) were lower than those of the grafting group (4.79± 0.46) and (2.56 ± 0.85), respectively (all p< 0.05). The wound healing time was positively correlated with pain score (r = 0.767, p< 0.05) and redness and swelling score (r = 0.672, p< 0.05), while negatively correlated with skin graft survival rate (r= -0.289, P<0.01), meeting linear equation y = -8.451 + 4.542a + 0.087b + 1.012c (a, pain score; b, skin graft survival rate; c, redness and swelling score).

**Conclusions:**

Punctate skin grafting combined with irrigation in the treatment of large-area residual burn wounds presents great effects on skin graft survival, redness and swelling score and pain, and is worthy of clinical application and promotion.

## INTRODUCTION

Patients with large area burn present irregular wounds, poor healing, repeated ulceration, edema, pain and even confusion, which seriously affect the therapeutic effect of the patients.^1^ In clinical practice, treatment methods for wound healing are diverse, mainly including wound immersion, irrigation, hydrotherapy and antibacterial disinfection.^2^ Although a certain therapeutic effect has been achieved, the skin graft survival rate in large-area wounds is not high.^3^ In clinic, large-area skin grafting is prone to falling off, redness, swelling and erosion, seriously affecting the therapeutic effect of patients and prolonging the wound healing time.^4^ How to accelerate the wound healing and improve the survival rate of skin grafts has become an important topic in the clinical research on burn treatment. In recent years, punctate skin graft has become a new method for treating large area burns, characterized by intensive skin grafting, shortening skin flap gap, reducing the exposure time of wounds and avoiding cross infection of wounds.^5^ To further improve the survival rate of skin grafts and weaken redness, swelling and pain, this paper advocates punctate skin grafting combined with irrigation in the treatment of large-area residual burn wounds. The report is as follows.

## METHODS

A prospective study was conducted on 102 patients with large-area burns treated in Huangshi Central Hospital from May 2017 to May 2020. According to different treatment methods, they were divided into combination group and grafting group, with 51 patients in each group. The differences between the two groups were not significant in general data such as gender, age and type of burns (*p>*0.05), suggesting comparability, as shown in Table-I.

**Table I:** Comparison of general data between two groups.

Group	N (Cases)	Male/female (n)	Age (year)	Type of burns		

				Flame burn	Molten steel burn	Chemical burn
Combination group	51	37/14	37.25 ± 5.36	24	26	1
Grafting group	51	36/15	36.84 ± 5.12	23	26	2
χ^2^/t	-	0.048	0.395		0.355	
P	-	0.826	0.694		0.838	

### Ethical Approval

The study was approved by the Institutional Ethics Committee of Huangshi Central Hospital, Affiliated Hospital of Hubei Polytechnic University on July 15, 2020 (Ref. No.: 2020035), and written informed consent was obtained from all participants.

### Inclusion Criteria:

- Meeting the diagnostic criteria of the Diagnostic Criteria and Treatment Guidelines for Burn Infection (2012 Edition) compiled by the Editorial Committee of the Guidelines for Diagnosis and Treatment of Burn Infection of the Burn Doctor Branch of the Chinese Medical Doctor Association;
- Burn area > 50%;
- With residual wounds after treated in Huangshi Central Hospital;
- Receiving no other treatment before the experiment;
- With the informed consent for the experimental study from the patients and their families.

### Exclusion Criteria:

- Patients with severe mental disease;
- Patients with severe infection;
- Patients with systemic skin tumors;
- Patients with congenital heart disease, acute myocardial infarction or arhythmia;
- Patients aged < 18 years.

The combination group was treated with punctate skin grafting combined with irrigation. The residual wounds were irrigated daily three days before surgery to keep clean. On the second day after surgery, the wounds were irrigated for 3-5 consecutive days, and then the next day. Irrigation was performed daily or every other day mainly using normal saline to help patients remove wound secretions and necrotic tissues. After preoperative dressing changes and irrigation, punctate skin grafting was carried out. Under an aseptic environment, autogenous thin skins were collected from the patients and cut into small rectangular punctate skin grafts with a size of 0.5 cm × 0.3 cm for intensive wound grafting, with the interval controlled within 0.5 cm. Finally, the grafts were fixed with 0.4% gentamicin gauze and wet bandages, and then bandaged with sterile gauze. The grafting group was mainly treated with punctate skin grafting. Before and after surgery, no standardized irrigation was performed, and the dressings were changed every five days. In addition, nutritional support was given to the two groups, and the needed nutrients and trace elements were supplemented in time.

### Evaluation Indexes

The positive rate of bacterial culture of wound secretions was analyzed and compared between the two groups. The positive rate of wound secretion culture was evaluated one week after treatment. Pain was compared between the two groups using the visual analogue scale after treatment, scoring 0-10. The higher the score, the stronger the pain. The redness and swelling scores of the two groups were compared, with scores of 0, 1, 2 and 3 from mild to severe, successively. Additionally, linear logistic analysis of the influencing factors was carried out.

### Statistical Methods

The data were statistically analyzed using SPSS 25.0. The enumeration data were expressed as n, % and analyzed by the chi-square test. The measurement data were expressed as mean ± standard deviation (*x̅*±*S*) and analyzed with the t-test. The correlation between each index was analyzed using Pearson’s correlation analysis. *P<* 0.05 was considered as statistically significant. In addition, each index was analyzed by linear logistic regression.

## RESULTS

Before treatment, no statistically significant difference was found in the positive rate between the combination group and the grafting group (*p>* 0.05). After treatment, the positive rate showed a statistically significant difference between the two groups (*p<* 0.05). In the combination group, a statistically significant difference was found in the positive rate before and after treatment (*p<* 0.05). In the grafting group, the positive rate also presented a statistically significant difference before and after treatment (*p<* 0.05) (Table-II).

**Table II:** Comparison in positive rate of bacterial culture of wound secretions between two groups before and after treatment.

Group	n	Before treatment	After treatment	t	*P*
Combination group	51	47 (92.2%)	15 (29.4%)	69.176	0.000
Grafting group	51	46 (90.2%)	25 (49.0%)	4.745	0.000
χ^2^		0.122	4.113		
P		> 0.05	< 0.05		

**Fig.1:**
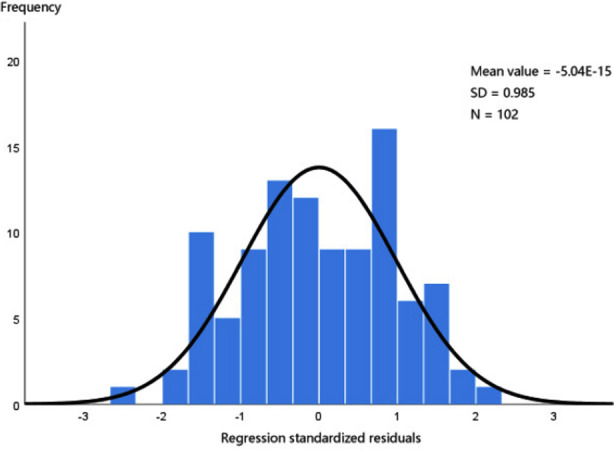
Histogram of linear logistic analysis of influencing factors.

In the combination group, the survival rate of skin grafts was higher, wound healing time was shorter, and pain score and redness and swelling score were lower than those in the grafting group (all *p<* 0.05). The wound healing time was used as the target variable, and the pain score, skin graft survival rate and redness and swelling score were taken as the independent variables, all of which were included into the hypothetical model, with the adjusted R-square value of 0.629 and the Durbin-Watson value of 2.481, meeting the requirements of modeling. The linear equation y **= -8.451 + 4.542a + 0.087b + 1.012c** (a, pain score; b, skin graft survival rate; c, redness and swelling score) was obtained. ANOVA showed that the F value was 57.973, which indicates the stability of the model, and *P* was 0.000 < 0.01, suggesting that pain score, skin graft survival rate and redness and swelling score can affect the wound healing time. From the correlation coefficients, wound healing time was positively correlated with pain score and redness and swelling score, while negatively correlated with skin graft survival rate, as seen in Table-IV. The constructed overall model conformed to the normal distribution, as shown in Fig.1, 2 & 3.

**Table III:** Comparison in skin graft survival rate, wound healing time, pain score and redness and swelling score between two groups.

	n	Skin graft survival rate (%)	Wound healing time (d)	Pain score	Redness and swelling score
Combination group	51	82.17 ± 7.44	14.56 ± 3.51	3.26 ± 0.36	1.16 ± 0.68
Grafting group	51	61.53 ± 7.46	21.36 ± 4.69	4.79 ± 0.46	2.56 ± 0.85
t		13.990	-8.290	-18.715	-9.188
P		< 0.01	< 0.01	< 0.01	< 0.01

**Table IV:** Correlation analysis of key indexes between two groups.

Index	Parameter	Wound healing time	Skin graft survival rate	Pain score	Redness and swelling score
Wound healing time	r	1	-0.289**	0.767**	0.672**
	*P*	-	< 0.01	< 0.01	< 0.01
	n	102	102	102	102
Skin graft survival rate	r	- 0.289**	1	-0.574**	-0.365**
	*P*	< 0.01	-	< 0.01	< 0.01
	n	102	102	102	102
Pain score	r	0.767**	-0.574**	1	0.745**
	*P*	< 0.01	< 0.01	-	< 0.01
	n	102	102	102	102
Redness and swelling score	r	0.672^**^	-0.365^**^	0.745^**^	1
	*P*	< 0.01	< 0.01	< 0.01	-
	n	112	112	112	102

** .*p<* 0.01, significant correlation.

**Fig.2:**
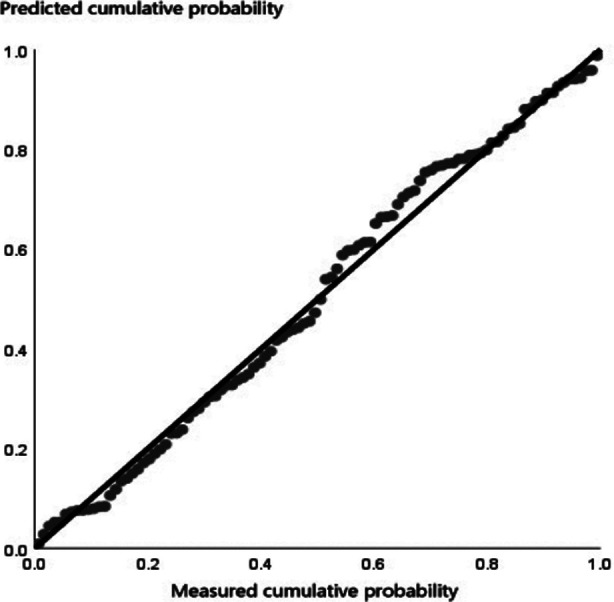
P-P diagram of linear logistic analysis of influencing factors.

**Fig.3:**
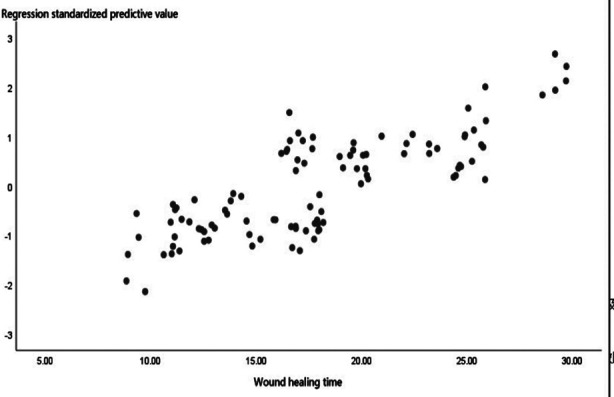
Scatter plot of linear logistic analysis of influencing factors.

## DISCUSSION

During the late treatment of patients with large area burns, some wounds will present a patchy pattern, easy infection and ulceration, and difficult automatic healing.^6^ In clinical practice, this problem is usually solved using combined treatment, that is, the combination of nutrition support, drug therapy and debridement of wound secretions, but the obtained therapeutic effect is not satisfactory.^7^ For patients, punctate skin grafting is performed by grafting a healthy thin skin onto the wound, which not only is simple in operation and can promote wound healing, but also can restore the appearance of the wound.^8^ This is different from large-area skin grafting in that the thin skin is cut into rectangular pieces of 0.3-0.5 cm and attached to the wound in time. The skin graft has low requirement in its growth, and obvious effect on wound healing. From the perspective of patients with large area burns, the application of punctate skin grafting can shorten treatment duration, prevent wound infection and avoid leaving scars. However, based on clinical practice, after partial punctate skin grafting, patients will present a large number of secretions within five days, and the skin graft is vulnerable to the erosion of fungi, bacteria and other microorganisms, leading to wound tears and even falling off, which weaken the therapeutic effect. Some studies have pointed out that irrigation using normal saline can remove postoperative secretions, reduce bacterial infection, and improve wound repair. For instance, Yao Miao and Chai Xuejun (2018) have pointed out that irrigation can remove wound secretions and necrotic tissues, prevent wound effusion and accelerate wound healing, and it is a new technology for clinical surgical treatment of wounds, which is suitable for clinical application. Some foreign medical research has also pointed out the necessity of irrigation for large area burns.^9^ Studies have shown that irrigation with normal saline and electrolyzed strong acid aqueous solution could induce epithelialization earlier, proliferate lymphocytes and macrophages, and promote the tissue regeneration of burn wounds, which has positive significance for the rehabilitation of patients.^10^

In the early stage of treatment, the positive rate of the combination group and the grafting group both reached more than 90%, and decreased to 29.4% after combined surgical treatment in the combination group, which is consistent with the study of Liu Wei et al. (2017).^11^ This fully demonstrates the significant effect of combined treatment on large area burns. The reason lies in that irrigation can avoid the accumulation of wound secretions and bacteria, reduce the formation of microbial communities, keep the wounds clean, promote the proliferation of epithelial cells, increase the adhesion of skin grafts and improve the survival rate of skin grafts. In terms of survival rate, the survival rate of the combination group was higher than that of the grafting group by nearly 20%, which has resulted from the decrease of blood loss, avoidance of bacterial adherent reproduction, reduction of cross infection, and improvement of the survival rate of burned tissues by irrigation. The improvement in tissue survival rate leads to the shortening of wound healing time, which is the reason why the wound healing time of the combination group was shorter than that of the grafting group. Studies have pointed out that infection and hematoma are the main causes of grafting failure, which need to be paid attention to^12^. Comparison of the redness and swelling data revealed that the redness and swelling of the combination group after treatment was significantly milder than that of the grafting group. According to the redness and swelling score, the difference was statistically significant (*p<* 0.05). This may be caused by that irrigation with normal saline, salt solution and liquor can not only remove bacteria and secretions, but also quickly reduce the local temperature at wounds, relieve pain, eliminate tissue edema, decrease lactic acid and histamine production, change the microcirculation and promote wound recovery. Moreover, it has been pointed out that cryotherapy is an effective method for treating burn wounds, which can accelerate wound healing and reduce infection rate if applied properly. Singer A J pointed out through a clinical study and statistical analysis that the initial pain score of burns was (6.3 ± 0.27).^13^ In this study, after treatment, the pain score of both groups decreased, but the score of the combination group was significantly lower than that of the grafting group. As for the causes, irrigating drugs can weaken the continuous damage of residual heat at wounds to active tissues, alleviate the metabolism of wound tissues, reduce the oxygen consumption of wound tissues, and have a significant analgesic effect.^14^

From the perspective of correlations, wound healing time, pain score, redness and swelling score and skin graft survival rate were significantly pairwise correlated (*p<* 0.05). Wound healing time, pain score and redness and swelling score were positively pairwise correlated, while skin graft survival rate was negatively correlated with other indexes. Tran S et al. have pointed out that pain is known to cause delayed wound healing.^15^ Our experimental study showed that the longer the pain time, the longer the wound healing time. They were positively correlated, which coincides with the study of Chester SJ et al., indicating that pain can also be used as an index to determine the degree of large-area wound healing.^16^ The reason may be that the pain of patients with large area burns comes from neuropathic pain, tissue injury pain, etc. which excite the autonomic nerve and stimulate the hypothalamus-pituitary-adrenal gland axis of the patients, leading to the rapid rise of cholesterol in the body and local hypoxia at the wounds, which affect the wound healing of the patients.^17^ For burn pain, a study has pointed out that psychological intervention and music therapy can alleviate pain, relieve sensory nerves and accelerate wound healing of patients.^18^ Redness, swelling and edema are also symptoms of patients with large area burns.^19^ After burned, central coagulation zone, middle stasis zone and peripheral hyperemia zone well appear on the injured surface. In addition, due to increased vascular permeability, plasma osmotic pressure will increase, finally leading to local swelling and edema. Moreover, it has been demonstrated that wound edema can be treated by compression and drug therapy, which can effectively alleviate the wound edema of patients.^20^

### Limitations of this study

It includes small sample size,short follow-up time, and failure to divide and study the post-operative pathological types, therapeutic effects and prognosis of patients in a more detailed manner due to small sample size. In view of this, proactive countermeasures will be taken in the future to carry out more comprehensive studies on such patients, so that more scientific data can be made available to our clinicians.

## CONCLUSION

In conclusion, combined treatment can directly improve skin graft survival rate, and eliminate redness, swelling and pain, laying good foundation for the rehabilitation of patients. Punctate skin grafting combined with irrigation in the treatment of large-area residual burn wounds presents great effects on skin graft survival, redness and swelling score and pain, and is worthy of clinical application and promotion.

### Authors’ Contributions:

**LW** & **LL:** Designed this study and prepared this manuscript, and are responsible and accountable for the accuracy or integrity of the work.

**JZ:** Collected and analyzed clinical data.

**JZ:** Significantly revised this manuscript.
